# Are *n*-3 PUFAs from Microalgae Incorporated into Membrane and Storage Lipids in Pig Muscle Tissues?—A Lipidomic Approach

**DOI:** 10.1021/acsomega.2c02476

**Published:** 2022-07-08

**Authors:** Dirk Dannenberger, Anja Eggert, Claudia Kalbe, Anna Woitalla, Dominik Schwudke

**Affiliations:** † Lipid Metabolism and Muscular Adaptation Workgroup, Research Institute for Farm Animal Biology, Institute of Muscle Biology and Growth, 18196 Dummerstorf, Germany; ‡ Institute of Genetics and Biometry, Research Institute for Farm Animal Biology, 18196 Dummerstorf, Germany; § Division of Bioanalytical Chemistry, Research Center Borstel—Leibniz Lung Center, 23845 Borstel, Germany; ∥ German Center for Lung Research (DZL), Airway Research Center North (ARCN), Research Center Borstel—Leibniz Lung Center, 23845 Borstel, Germany; ⊥ German Center for Infection Research, Thematic Translational Unit Tuberculosis, Research Center Borstel—Leibniz Lung Center, 23845 Borstel, Germany

## Abstract

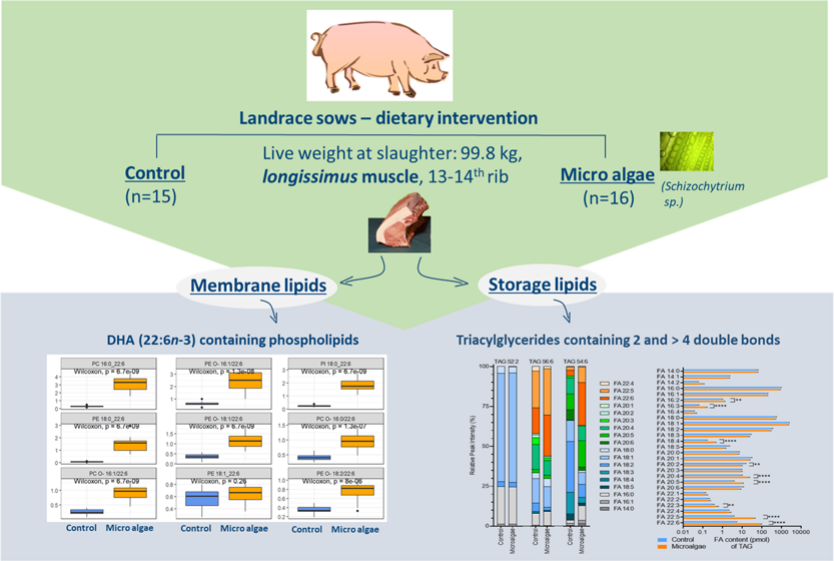

For the study of molecular mechanisms of to lipid transport and storage in relation to dietary effects, lipidomics has been rarely used in farm animal research. A feeding study with pigs (German Landrace sows) and supplementation of microalgae (*Schizochytrium* sp.) was conducted. The animals were allocated to the control group (*n* = 15) and the microalgae group (*n* = 16). Shotgun lipidomics was applied. This study enabled us to identify and quantify 336 lipid species from 15 different lipid classes in pig skeletal muscle tissues. The distribution of the lipid classes was significantly altered by microalgae supplementation, and ether lipids of phosphatidylcholine (PC), phosphatidylethanolamine (PE), and phosphatidic acid (PA) were significantly decreased. The total concentration of triacylglycerides (TAGs) was not affected. TAGs with high degree of unsaturation (TAG 56:7, TAG 56:6, TAG 54:6) were increased in the microalgae group, and major abundant species like TAG 52:2 and TAG 52:1 were not affected by the diet. Our results confirmed that dietary DHA and EPA are incorporated into storage and membrane lipids of pig muscles, which further led to systemic changes in the lipidome composition.

## 1 Introduction

Dietary strategies to modify the fatty acid profile of pig muscles by enhancing polyunsaturated fatty acid (PUFA) contents, predominantly *n*-3 PUFA, were very successful. In contrast to the ruminants, it was shown in the pigs that dietary PUFAs could be incorporated into muscles and other tissues with only minor biochemical modifications of PUFAs. Intervention studies on PUFA-supplemented pig diets containing linseed, rapeseed, sea buckthorn, pomace cakes/oils/meals resulted in an increase in *n*-3 PUFA content and a decrease in the *n*-6/*n*-3 fatty acid ratio in intramuscular fat.^1−4^ In contrast, sunflower seed/oil/meal-supplemented diets revealed an increase in *n*-6 PUFA content in pig muscle tissues.^5^ The supplementation with microalgae in the pig diet is a potential alternative to improve the lipid/fatty acid profile of pig muscle with respect to human nutrition compared to vegetable oils/press cakes/meals.^6−8^ Besides essential amino acids, vitamins, polysaccharides, microalgae contain long-chain *n*-3 PUFAs, primary docosahexaenoic acid (C22:6*n*-3, DHA) or eicosapentaenoic acid (C20:5*n*-3, EPA). A large amount of evidence suggests that EPA and DHA have stronger beneficial effects on human health compared to C18:3*n*-3. Furthermore, the conversion of C18:3*n*-3 to EPA and DHA is limited in humans (<10%); therefore, direct consumption of foods rich in EPA and DHA is required to reach the recommended daily *n*-3 PUFA intake.^6,7^

First lipidomic approaches were performed in pigs and beef cattle primarily using electrospray ionization-tandem mass spectrometry (UHPLC-ESI-MS/MS), shotgun lipidomics, or MALDI-TOF MS.^9−13^ Only a small number of lipidomic studies on pig muscles are available. Recently, lipidomics was applied to identify different muscles of pigs and to differentiate/authenticate raw pork species. The comparative lipidomic analysis of selected local pork in China led to the definition of a lipid marker panel that could classify different pork cuts and geographical origins.^14^ Another study with a targeted lipidomic approach using HPLC-ESI-MS/MS was conducted to investigate pig muscle phospholipids (PLs) and variations of phospholipid hydrolysis products at different aging periods.^15^ As the main phospholipid classes in pig muscles, phosphatidylcholine (PC), ether-linked PC (PC-O), phosphatidylethanolamine (PE), and ether-linked PE (PE-O) were identified as comprising up to 70% of the overall PL content.^15^

Very recently, Meyer et al.^13^ investigated the replacement of soybean extraction meal with insect meal in the diet of growing pigs using transcriptomics, metabolomics, and lipidomics. A 4 week insect meal-based diet (*Tenebrio molitor* L.) in growing pigs revealed only weak changes in the lipid metabolism in the plasma and liver. The concentrations of main PL classes, such as PC, PE, phosphatidylinositol (PI), lysophospholipids, and sphingolipids, were not affected by the insect-based diet; however, no further lipid analysis of pig muscle tissue was performed. The first study investigating the changes of lipid profiles in skeletal muscle of Landrace pigs fed with *n*-3 or *n*-6 PUFA-rich diets indicated large differences between the diet groups.^10^ The results showed that dietary and de novo synthesized *n*-3 PUFAs were predominantly incorporated into muscle PL species PE and cardiolipins (CL); however, the distribution pattern of different PL classes in pig muscle was unchanged. In addition, alkenyl-acyl and alkyl-acyl phospholipids (ether-linked PLs) were elevated in muscle of pigs fed with *n*-3 PUFA-based diets.^10^ The occurrence, molecular, and physiological impact of ether-linked PLs, primarily plasmalogens, in pig tissues is still enigmatic.^15^

In general, the application of microalgae as a diet supplement for pigs opens up the opportunity to improve growth and meat quality in pigs and also in ruminants; however, the results are affected by the supplemented microalgae species.^6^ Recently, our group investigated the effects of long-term microalgae supplementation (*Schizochytrium* sp.) on muscle microstructure, meat quality, and fatty acid composition in growing German Landrace pigs.^8^ The samples of pig muscles, highly accumulated in docosahexaenoic acid (DHA) and eicosapentaenoic acid (EPA), were used in this nontargeted shotgun lipidomic study. The objective of the present study was to investigate which lipid species predominantly influence the dietary changes with increased levels of long-chain *n*-3 PUFAs in pig skeletal muscle (longissimus thoracis) and their incorporation in membrane and/or storage lipids by dietary microalgae supplementation.

## 2 Experimental Section

### 2.1 Animal Study Design and Sample Collection

The study was conducted at the experimental pig facilities of the Research Institute for Farm Animal Biology (FBN) in Dummerstorf, Germany. All procedures, including the use and treatment of animals, were performed in accordance with the German animal protection law and approved by the relevant authorities (Landesamt für Landwirtschaft, Lebensmittelsicherheit und Fischerei Mecklenburg-Vorpommern, Germany; 7221.3-2-051/15). The experimental details of the dietary study of microalgae supplementation on meat quality and muscle microstructure in growing pigs were recently described.^8^ Briefly, the dietary pig study (German Landrace sows) was performed with the supplementation of microalgae (*Schizochytrium* sp.). The piglet diet was fed until day 95 (∼13.7 MJ of ME/kg) and followed the fattening diet (∼12.9 MJ of ME/kg). The microalgae (*Schizochytrium* sp.) diet was supplemented with 7% (piglet diet) and 5% (fattening diet) DHA Gold (DSM, Bramsche, Germany), whereas the control diet was adjusted to a total fat of 5.4% (piglet diet) and 3.2% (fattening diet) using soybean oil plus lard as lipid sources. The major difference between diets consisted in the proportion of DHA (37% in the piglet diet and 33% in the fattening diet of the microalgae group and 0.05% in the piglet diet and 3.1% in the fattening diet of the control group).^8^ The female piglets were allocated to the control group (*n* = 15) or the microalgae group (*n* = 16) at day 28 of age. After one week of adaptation, the microalgae-supplemented diet was initiated on day 35 of age and was fed until pigs were slaughtered at days 145/146 of age. After slaughtering, muscle samples were immediately collected from the right side of the carcass. Longissimus thoracis (LT) muscle samples of pigs obtained from the 12/14th rib were used for lipidomic investigation.

### 2.2 Lipid Extraction from Pig Muscles

Immediately after sampling, the muscle samples were cut into small pieces, deep-frozen in liquid nitrogen, and homogenized under liquid nitrogen using a stainless steel grinding mill (mill M20, IKA, Staufen, Germany). After homogenization, the muscle samples were stored at −80 °C until total lipid extraction. The total lipids of muscle samples (2 g muscle powder) were extracted using an Ultra Turrax (T25, IKA, Staufen, Germany), 3 × 15 s, 15,777*g* using chloroform/methanol (ratio 2:1) at room temperature. All solvents contained 0.005% (w/v) of *t*-butylhydroxytoluene (BHT) to prevent PUFA oxidation. The details of the lipid extraction procedure were already described.^8^ The final extraction mixtures were stored at 5 °C for 18 h in the dark and subsequently washed with a 0.02% CaCl_2_ solution. The organic phase was separated and dried with a mixture of Na_2_SO_4_ and K_2_CO_3_ (10:1, w/w), and the solvent was subsequently removed using a ScanSpeed 40 (LaboGene, Allerød, Denmark) vacuum centrifuge at 438*g* and 30 °C for 30 min. In total, 31 pig muscle lipid extracts (control group, *n* = 15; and microalgae group, *n* = 16) were stored at −20 °C until lipidomic analysis.

### 2.3 Shotgun Lipidomics

The muscle lipid extracts were dissolved in chloroform/methanol/water (60:30:4.5, v/v/v) with BHT at 0.05% (w/v). The individual samples were in a second dilution step normalized to 8.38 mg/mL of total fat. Afterward, all samples were mixed with an internal standard solution (Supporting Table S1) and the ESI spray solution as reported earlier with a final dilution factor of 1100 from the stock solution.^16^ Shotgun lipidomics was performed by a Q Exactive Plus (Thermo, Bremen, Germany) using TriVersa NanoMate (Advion, Ithaca) as a nanoelectrospray source as reported earlier.^16^ For lipid identification, LipidXplorer 1.2.7 was utilized, and quantification was achieved using responses of the respective lipid class-specific standard as reported earlier.^16−18^ Lipidomic data processing details are available on the Lipidomics Informatics for Life Science (LIFS) web portal (https://lifs-tools.org/).^19^ Cholesterol concentrations were determined based on an approach reported earlier.^20^ The reported lipidomes will be made available under the preliminary LipidCompass accession number LCE9.

All used solvents and chemicals were obtained in the highest purity grade (ROTISOLV, HPLC grade) from Carl Roth GmbH (Karlsruhe, Germany). For lipidomic experiments, all used solvents and additives were of LC–MS quality and obtained from Sigma-Aldrich (Deisenhofen, Germany). Lipid standards were purchased from Avanti Polar Lipids (Alabaster).

### 2.4 Data Analysis and Statistics

A total of 336 lipid species quantified using shotgun lipidomics were grouped in samples of the control group (*n* = 15) and microalgae group (*n* = 16). Multiple *t*-tests between control and microalgae groups were performed using log-transformed data. To adjust for multiple comparisons, we calculated *q*-values and limited the false discovery rate to 0.01 using the *R* package *q* value (v2.22.0^21^). In addition, the log_2_-fold change (log_2_FC) between microalgae and control lipid species concentrations (nmol/mg total lipids) was calculated to quantify the variations.

We applied partial least-squares discriminant analysis (PLS-DA) to identify the key variables of the 15 lipid classes (including two subclasses) and the sparse variant (sPLS-DA) for the 336 lipid species that drive the discrimination of the two investigated groups, i.e., control vs microalgae-supplemented group. Both methods are implemented in the mixOmics package (v6.14.1^22^). We used scaled data to analyze the lipid classes, while the individual lipid species were not scaled as we aimed to keep the information of the lipid concentration and distinguish between the relevance of minor and major lipids. Sample plots are presented to visualize the discriminatory ability of the lipid classes and individual lipid species in the space spanned by the first two latent variables. Loading plots show the importance of the 15 lipid classes and 15 lipid species, which had the strongest impact on group separation in PLS-DA or sPLS-DA, respectively. We used repeated 5-fold cross-validation to evaluate the performance of the fitted PLS-DA models. The models have a very good performance in discriminating the two treatment groups with a stabilized balanced error rate of 0.015 (lipid classes) and an error rate close to zero (lipid species) after two components. Statistical data analyses and data visualization were performed using R 4.0.3 (R Core Team, 2020).^21^

## 3 Results

In this study, 336 lipids of 15 different classes were identified and quantified in total lipid extracts of skeletal muscle tissues of pigs fed either with a control died or supplemented with microalgae (Supporting Table S2). The highest number of species in pig muscle extracts were identified for TAGs (57 lipid species), alkyl/alkenyl-phosphatidylethanolamines (PE-O, 52 lipid species), PEs (47 lipid species), alkyl/alkenyl-phosphatidylcholines (PC-O, 43 lipid species), and PCs (30 lipid species). The concentration range for a single lipid covered more than 5 orders of magnitude with TAG 52:2 as the major abundant component (210 nmol/mg total lipids) and LPE 22:6 (0.002 nmol/mg total lipids, Supporting Table S2). TAG was found as the most abundant lipid class with 65–68% of total lipids in a concentration of 833.3 ± 75.3 nmol/mg total lipids (control group) and 871.9 ± 49.8 nmol/mg total lipids (microalgae group). The overall TAG content was not affected by microalgae supplementation. In the case of membrane lipids, our lipidomic analysis demonstrated that PC and PE (including the ether-linked PLs—PC-O and PE-O) were the most abundant PL classes and represent approximately 80% of total PL content independent of the dietary intervention. Both subclasses PC-O and PE-O were significantly reduced by supplementation with microalgae (Table 1). Furthermore, phosphatidic acid, comprising only two quantifiable lipid species, showed significantly lower concentration in the microalgae group with 0.38 ± 0.03 nmol/mg total lipids. No further changes were observed for the remaining lipid classes (Table 1). It is further noteworthy that free cholesterol levels were not altered due to microalgae supplementation (Supporting Table S2).

**Table 1:** Mean Sum Concentrations and Standard Error of the Mean (SEM) of 15 Lipid/Phospholipid Classes (nmol/mg Total Lipids) in Muscles (M. Longissimus) of Pigs Fed with Control vs Microalgae-Based Diet, Adjusted *p*-Values of Multiple *t*-Tests Using log-Transformed Data by the Benjamini-Hochberg-Procedure, and log_2_-Fold Changes^a^

		control group	microalgae group			
lipid class^b^	number of lipid species	mean ± SEM (*n* = 15)	mean ± SEM (*n* = 16)	adjusted *p*-value	log_2_-fold change	significant differences
TAG	57	833.3 ± 75.3	871.9 ± 49.8	0.559	0.07	no
PC	30	127.2 ± 7.4	146.8 ± 9.5	0.333	0.21	no
PC-O	43	97.2 ± 5.2	50.9 ± 3.1	<0.001	–0.93	yes
PE-O	52	74.8 ± 4.0	52.6 ± 3.2	0.002	–0.51	yes
PE	47	41.7 ± 2.4	45.5 ± 3.0	0.559	0.13	no
SM	9	33.9 ± 2.1	36.8 ± 2.8	0.648	0.12	no
PI	28	31.7 ± 2.9	27.0 ± 2.0	0.483	–0.23	no
PS	16	21.6 ± 1.4	17.3 ± 1.0	0.108	–0.32	no
LPI	10	9.0 ± 0.7	8.0 ± 0.9	0.426	–0.16	no
DAG	12	6.8 ± 0.6	6.4 ± 0.8	0.648	–0.08	no
LPC	10	5.5 ± 0.7	8.5 ± 1.4	0.171	0.65	no
LPE	10	1.0 ± 0.1	1.4 ± 0.2	0.333	0.43	no
CL	3	1.0 ± 0.1	1.0 ± 0.1	0.834	0.06	no
PA	2	0.65 ± 0.04	0.38 ± 0.03	<0.001	–0.77	yes
PG	4	0.17 ± 0.02	0.09 ± 0.05	0.430	0.04	no

a Differences are classified as being significant and have a type 1 error *p* < 0.05 and a log_2_-fold change > 0.5.

b Abbreviations of lipid classes: triacylglyceride, TAG; phosphatidylcholine, PC; ether-linked PC, PC-O; ether-linked PE, PE-O; phosphatidylethanolamine, PE; sphingomyelin, SM; phosphatidylinositol, PI; phosphatidylserine, PS; lyso-phosphatidylinisitole, LPI; diacylglyceride, DAG; lyso-phosphatidylcholine, LPC; lyso-phosphatidylethanolamine, LPE; cardiolipin, CL; phosphatidic acid, PA; phosphatidylglycerol, PG.

**Figure 1:**
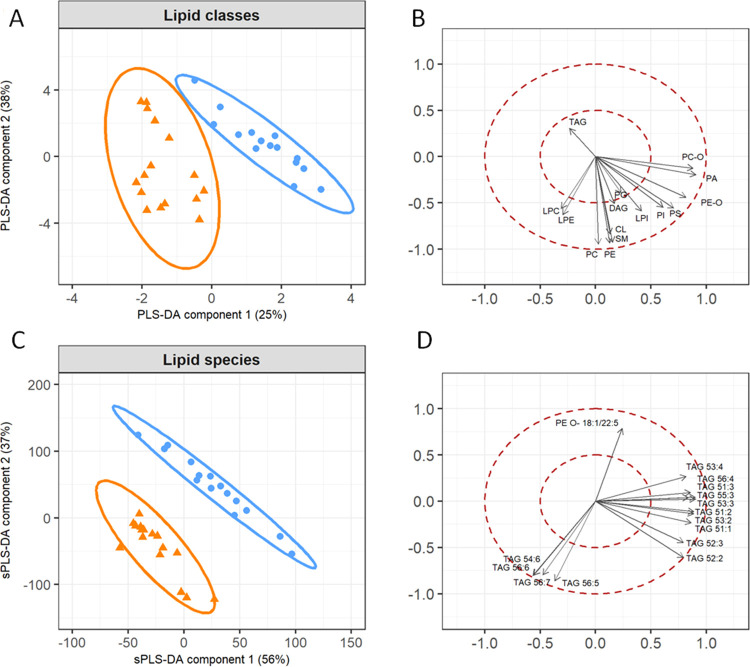
Partial least-squares discriminant analysis (PLS-DA) for the lipidome of pig muscle tissue (longissimus thoracis). (A, C) PLS-DA sample plot and loading plot for the total concentration of the analyzed 13 lipid classes and 2 subclasses and (B, D) PLS-DA sample plot and loading plot for the concentration of 15 important lipid species, referring to a cutoff of 0.785. The data set comprised lipidome data of the control group (*n* = 15—blue) and microalgae group (*n* = 16—orange). In the loading plot (D), 15 lipid species were selected, which had the strongest impact on group separation (the complete data set is listed in Supporting Table S2).

To gain further insight into the metabolic imprint caused by microalgae supplementation, partial least-squares discriminant analysis (PLS-DA) was applied (Figure 1).

The PLS-DA analysis based on concentrations of lipid classes already revealed a clear separation of the two diet groups (Figure 1A). However, the analysis of the 336 lipid species in muscles of both diet groups (Figure 1C) resulted in a much clearer separation. This analysis indicated that PLS-DA with all species in the muscle of the two groups provides a much more differentiated separation of the two groups when comparing the sum concentration of the lipid classes. This is an indication that specific lipid species were strongly affected by the diet. In addition, the loading plots of total concentrations of lipid classes (Figure 1B) and concentrations of 15 important lipid species (Figure 1D) (referring to a cutoff of 0.785) were presented. This analysis further underlined the strong influence of TAG species on the separation of the diet groups shown in PLS-DA (Figure 1C). TAGs with a high degree of unsaturation were increased in the microalgae group, which were in contrast with some of the major abundant species like TAG 52:2 and TAG 52:1 that were not affected by supplementation. This illustrates that the use of all 336 single lipid species identified in pig muscle for PLS-DA lead to a stronger separation of both diet groups compared to the use of 15 lipid classes, only. Further pairwise comparison of the lipidomes revealed that 199 out of 336 lipid species showed significant concentration changes that represent a prevalence of 66% (*q* < 0.01).

**Figure 2:**
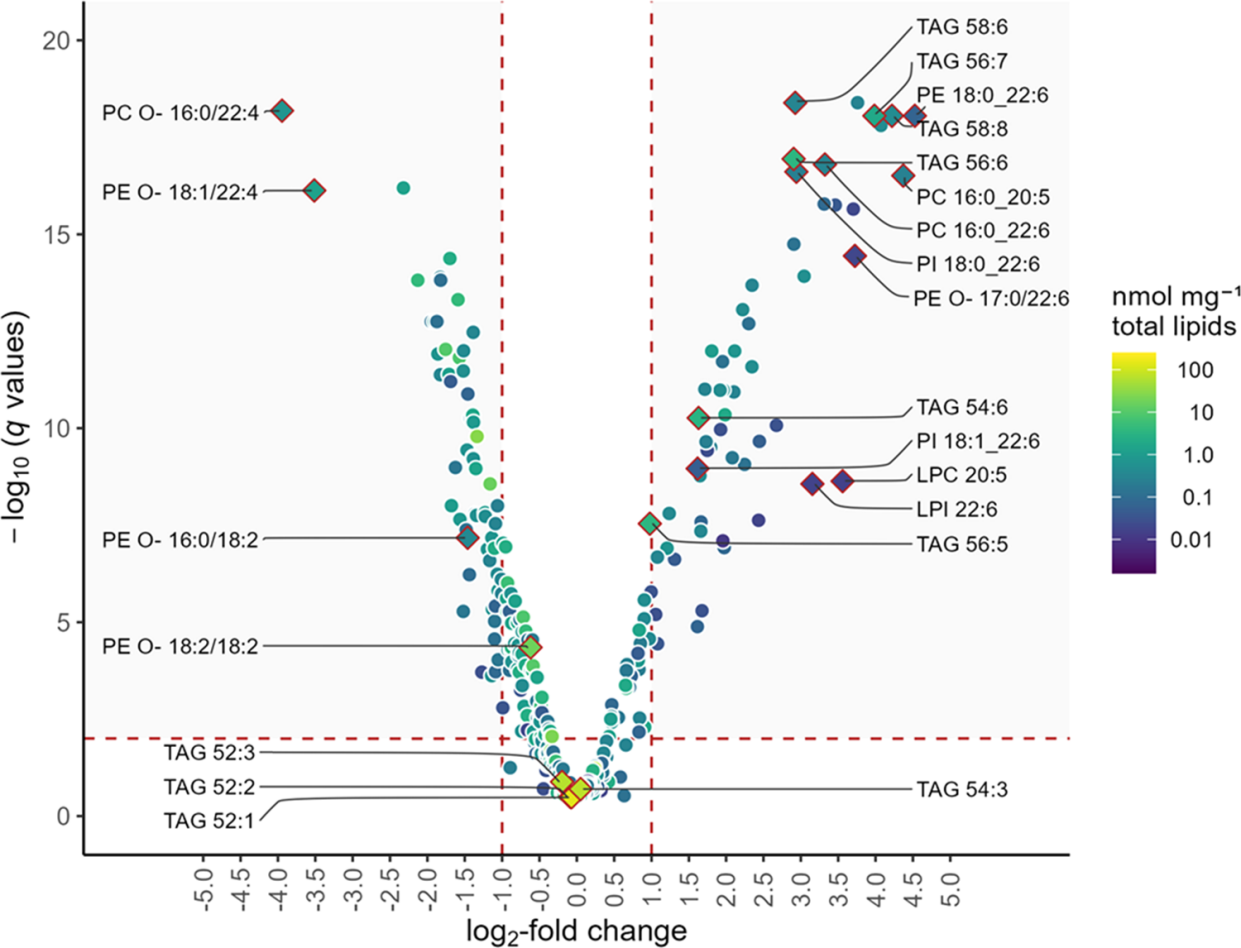
Comparison of altered lipid quantities in muscle tissue (longissimus thoracis) of pigs fed according to the control diet and with microalgae supplementation. *q*-values were calculated from *p*-values of multiple *t*-tests between control and microalgae groups using log-transformed data, limiting the false discovery rate to 0.01. Differences are classified as being substantial (gray shaded area) if they are both significant with *q* < 0.01 and have a log_2_-fold change > 1. Selected lipid species are annotated according to the complete list of quantified lipids (Supporting Table S2).

Using more stringent cutoff criteria, still 113 of the 336 lipid species (34%) showing significant differences (*q* < 0.01) also have substantial changes in concentration (abs [log_2_FC] > 1.0). This result was visualized in a volcano plot (Figure 2 and Supporting Table S2) and underlines that microalgae supplementation is strongly reflected in the muscle lipidome.

**Figure 3:**
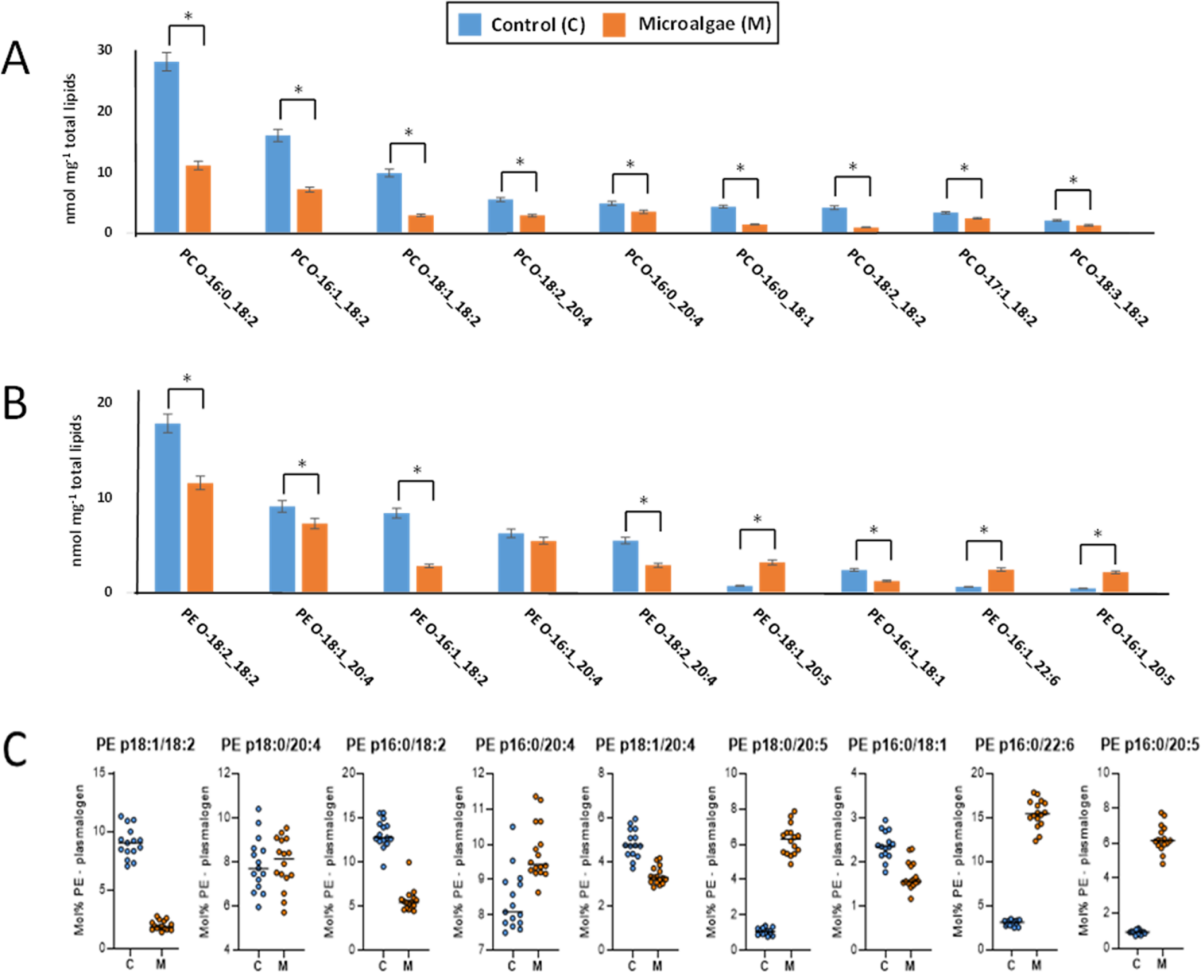
Concentrations (nmol/mg total lipid) of most abundant alkyl/alkenyl ether of PC-O (A) and PE-O (B) in muscle (longissimus thoracis) of pigs fed with control vs microalgae-based diet (*significant differences log_2_-fold change > 1, *q* value < 0.01), sorted by the highest mean concentrations. (C) PE-plasmalogen analysis derived from the positive ion mode tandem mass spectrometric analysis according to its specific fragmentation.^23,39^ The molar percentage was computed on basis of the PE-plasmalogen fragment ion intensities for all identified 49 identified species in control (C) and microalgae (M) groups (Supporting Table S3).

**Figure 4:**
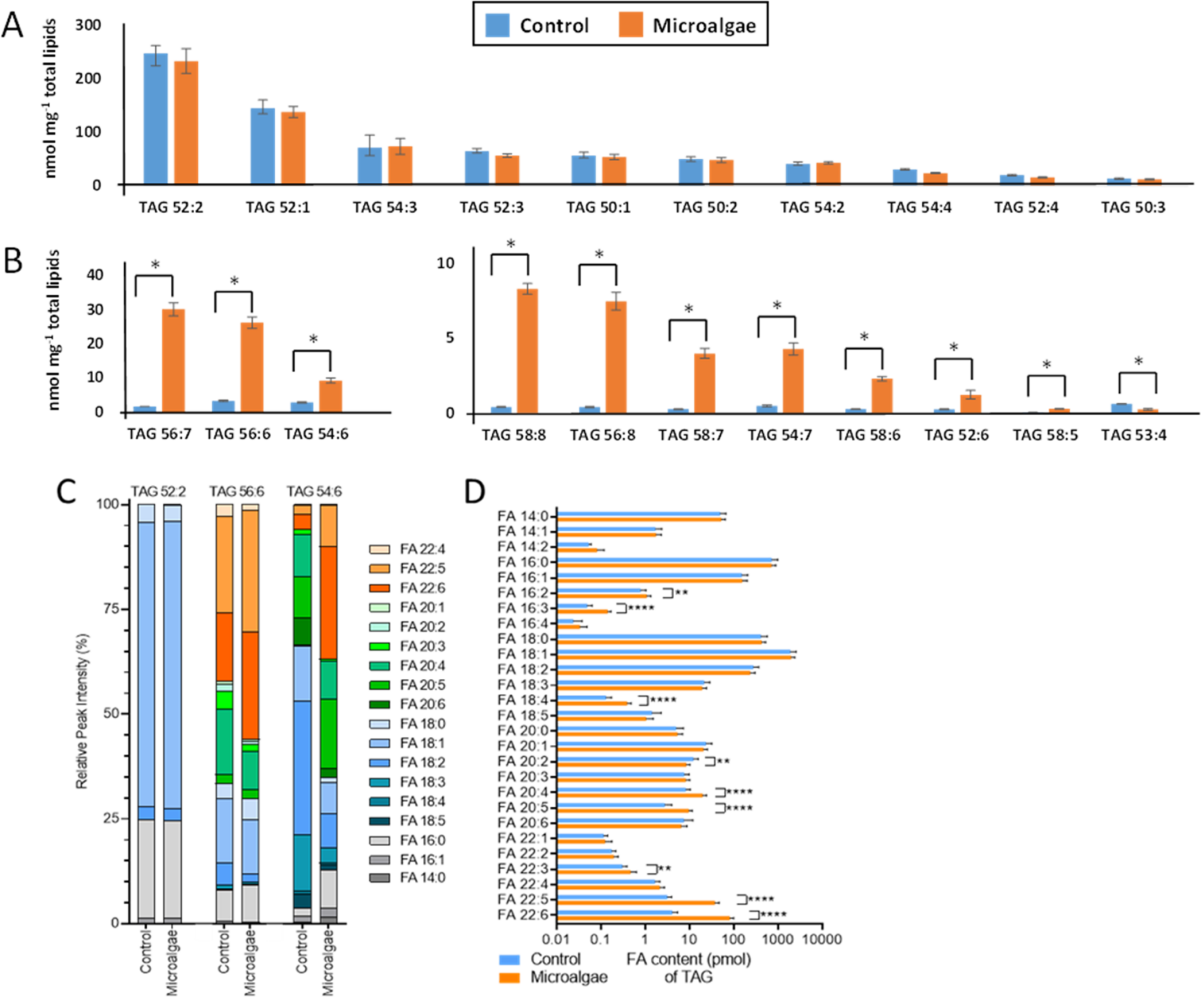
Concentrations (nmol/mg total lipid) of TAG species containing up to four double bonds (A) and ≥ 4 double bonds (B) in muscles (longissimus thoracis) of pigs fed with control vs microalgae-based diet (*significant differences log_2_-fold change > 1, *q* value < 0.01), sorted by the highest mean concentrations. (C) Analysis of the fatty acid composition of TAG 52:2, TAG 56:6, and TAG 54:6. [TAG + NH_4_]^+^ adduct ions undergo a neutral loss (NL) of the fatty acid and ammonia and the contribution of single fatty acids can be estimated from the intensities of resulting fragment ions (Supporting Figures SF1–SF4). (D) Overall FA content of TAG determined from MS^2^ analysis. Presented profiles are the mean values for the complete data set, control (*n* = 16) and microalgae (*n* = 15).

Next, we investigated in detail which lipids were mostly affected with a focus on two questions. First, do lipids comprising DHA and EPA show increased abundance after microalgae supplementation? Second, are there compensational effects detectable? For the top 20 nutritional most affected lipids, representing 63.5% of overall concentration change (Table 2), one can recognize that lipids with more than four double bonds were increased in abundance. The strongest gain in abundance was observed for highly unsaturated TAGs that are likely to comprise DHA and EPA, the main *n*-3 PUFAs in lipids of microalgae (*Schizochytrium* sp.). At the same time, it can be observed that the six PE-O and PC-O lipids of the top twenty list were decreased in concentration compared to the control (Table 2). Furthermore, it can be assumed that for all PE-O and PC-O species comprising aliphatic chains with double bonds ≤ 4 a reduction in concentration was observed (Figure 3A,B). Membrane lipids comprising DHA (*n* = 18/19) and EPA (*n* = 13/14) showed in the majority of cases increased abundance in the microalgae group. With particular interest, PE-O species using a specific mass spectrometric fragmentation mechanism for identification of PE-plasmalogens were analyzed.^23^ This analysis further supported the general observation that DHA and EPA comprising lipids were increased in concentration in the microalgae group, while main components with double bonds ≤ 4 were generally reduced when compared to the control (Figure 3C and Supporting Table S3). A detailed analysis of TAG profiles was performed, which confirmed that most of the abundant species with fewer double bonds were not affected by microalgae intervention. TAG 52:2 and TAG 52:1 in pig muscle had concentrations of 247 nmol/mg total lipids (control) vs 232 nmol/mg total lipids (microalgae) and 144 nmol/mg total lipids (control) vs 137 nmol/mg total lipids (microalgae), respectively (Figure 4A and Supporting Table S2).

**Table 2:** Mean Concentrations (nmol/mg Total Lipids) and Standard Error Mean (SEM) of the Top 20 Lipid Species in Muscles (M. Longissimus) of Pigs Fed with Control vs Microalgae-Based Diet According to Delta (Group Differences), log_2_-Fold Change between Mean Concentrations, Absolute Differences between Group Means, and *q*-Values Limiting the False Discovery Rate (FDR) to 0.01^a^

		control group	microalgae group			
no	lipid species	mean ± SEM (*n* = 15)	mean ± SEM (*n* = 16)	log_2_-fold change	Delta	*q* value (FDR-corrected)
1	TAG 56:7	1.9 ± 0.01	30.2 ± 1.9	3.98	28.31	8.83e^–19^
2	TAG 56:6	3.5 ± 0.2	26.3 ± 1.7	2.90	22.81	1.14e^–17^
3	PC-O-16:0/18:2	28.0 ± 1.5	11.1 ± 0.7	–1.33	16.90	1.63e^–10^
4	PC-O-16:1/18:2	16.0 ± 1.0	7.2 ± 0.4	–1.16	8.84	2.73e^–9^
5	TAG 58:8	0.45 ± 0.03	8.4 ± 0.4	4.22	7.90	8.83e^–19^
6	TAG 56:8	0.45 ± 0.04	7.5 ± 0.6	4.07	7.07	1.56e^–18^
7	PC-O-18:1/18:2	9.8 ± 0.6	2.9 ± 0.19	–1.76	6.95	9.21e^–13^
8	TAG 54:4	30.6 ± 2.0	24.0 ± 1.2	–0.35	6.64	9.13e^–3^
9	TAG 54:6	3.0 ± 0.2	9.4 ± 0.7	1.63	6.38	5.44e^–11^
10	PE-O-18:2/18:2	17.9 ± 1.0	11.6 ± 0.2	–0.62	6.24	4.45e^–5^
11	PC16:0_20:4	1.9 ± 0.2	7.6 ± 0.6	1.98	5.70	4.52e^–11^
12	PE-O-16:1/18:2	8.4 ± 0.5	2.8 ± 0.2	–1.57	5.58	1.51e^–12^
13	PC16:0_20:5	0.25 ± 0.02	5.1 ± 0.4	4.37	4.89	3.07e^–17^
14	PC16:0_20:5	22.8 ± 1.3	18.1 ± 1.0	–0.33	4.67	8.77e^–3^
15	TAG 54:7	0.52 ± 0.05	4.3 ± 0.4	3.04	3.80	1.20e^–14^
16	TAG 58:7	0.30 ± 0.02	4.0 ± 0.3	3.76	3.73	4.05e^–19^
17	PC 18:1_18:2	10.4 ± 0.6	6.9 ± 0.5	–0.58	3.48	1.33e^–4^
18	PS 18:2_18:0	8.7 ± 0.5	5.3 ± 0.3	–0.71	3.40	7.38e^–6^
19	TAG 56:5	3.5 ± 0.2	6.8 ± 0.3	0.97	3.36	2.89e^–8^
20	PC-O-18:2/18:2	4.2 ± 0.3	1.0 ± 0.05	–2.13	3.24	1.52e^–14^

a The table is sorted by the highest group differences.

They were not diet-affected, also reflected in the sum TAG concentrations (Table 1). However, 10 of the 57 TAG species (e.g., TAG 56:6, TAG 56:8, TAG 54:7, Figure 4B) comprising at least 5 double bonds showed significantly increased concentration with log_2_FC > 1. MS^2^ analysis of these TAG species revealed the increased incorporation of DHA and EPA for the microalgae group. This was exemplarily shown for TAG 56:6 and TAG 54:6 in Figure 4C, while the FA composition for TAG 52:2 was not changed. Subsequently, the FA compositional changes between both groups for all TAG species were determined (Figure 4D). Besides the expected increase for DHA and EPA, the significantly increased abundance of fatty acids (FAs) 16:2, 16:3, 18:4, 20:4, 22:3, and 22:5 could further be observed. Some of these FAs were not analyzed in standard assays, and at this level of analysis, the identity of isomers cannot be determined resulting from double bond position and configuration. Noteworthy is also the detection of the highly unsaturated FAs 20:6 and 18:5 that were not affected by microalgae intervention. Our results confirm that dietary DHA and EPA, highly enriched in lipids of microalgae (*Schizochytrium* sp.), were incorporated into storage and membrane lipids in pig muscles. Thus, supplementation with microalgae offers a unique opportunity to increase the levels of essential *n*-3 LC-PUFAs (DHA, EPA) in pork and thus contributes to the recommended intake of long-chain *n*-3 PUFAs by the consumers.

## 4 Discussion

Dietary *n*-3 PUFAs incorporated in complex lipids can affect a range of metabolic and physiological functions, such as energy storage, membrane organization, and signal transductions via lipid mediators synthesized by cyclooxygenase (COX), lipoxygenase (LOX), and cytochrome P450 (CYP). These physiological important processes are modulated in regard to different key lipids in which *n*-3 PUFAs are bound. Accordingly, lipidome analysis was performed to catalogue-induced compositional changes by *n*-3 PUFA supplementation in pig muscle tissues.^24^ It has been shown that dietary *n*-3 PUFAs can inhibit the transcription of lipogenic genes by suppressing sterol regulatory element-binding protein 1c (SREBP-1c) gene expression or by inhibiting the proteolytic release of nuclear SREBP-1c in pig muscles and adipose tissues.^25,26^ It appears that *n*-3/*n*-6 PUFAs act as ligands/modulators for nuclear receptors, thereby suppressing de novo fatty acid synthesis, and thus, lipogenesis and *n*-3 PUFAs appear to be more potent than *n*-6 PUFAs.^27^ Lipidomic approaches on pig muscle tissues to study dietary effects and lipid metabolism have so far only been conducted very rarely.^10^

Our present study using microalgae (*Schizochytrium* sp.) clearly demonstrated the incorporation of dietary DHA and EPA (up to 37% DHA and 1% EPA in total diets^8^) in membrane and storage lipids of pig muscle tissues (Table 2 and Supporting Table S2). Microalgae supplementation led to an almost two times higher number of lipid species containing ≥ 5 double bonds in pig muscles compared with the control. This incorporation of DHA/EPA should lead to the reorganization of membrane composition in pig muscles, which was indicated by the changed meat quality in our study as published before.^8^ Microalgae supplementation increased the water holding capacity (WHC) and the protein proportion in Landrace pig muscles compared to the control group.^8^ The DHA/EPA incorporation into membrane lipids could be predominantly shown for PC16:0_22:6, PE18:0_22:6, PI18:0_22:6, and EPA-containing species (PC16:0_20:5, PE18:0_20:5) with the highest species concentrations in muscle of pigs fed with microalgae-supplemented diets. This supports the hypothesis that primarily *n*-3 PUFAs seem to enable the muscle fibers to build a more flexible lipid bilayer membrane associated with higher WHC in pigs.^28^ Moreover, the observed compositional changes of membranes by the incorporation of EPA/DHA in skeletal muscle lipid species appear to stimulate the synthesis of proteins, resulting in higher total protein contents in pig longissimus thoracis muscle.^8^

DHA/EPA incorporation into the membrane lipid species was contrasted by the displacement of ether lipid species containing *n*-6 PUFAs with double bonds ≤4 in the acyl chains (e.g., PC-O-16:0_18:2, PC-O-16:1_18:2, PE-O-18:1_20:4, Figure 3), resulting in lower species concentrations. This is in line with our results of total fatty acid analysis showing lower 18:2*n*-6, 20:4*n*-6, and 22:4*n*-6 concentrations in muscles of pigs fed with microalgae compared to the control group.^8^ This finding suggests that systemic changes have occurred because of nutritional intervention with microalgae. Generally, it is known that ether-linked phospholipids represent about 20% of total PLs in mammalian cells.^15^ These ether lipids comprise two structurally different types, defined by either alkyl or alkenyl linkage of the aliphatic chain on glycerol, which have different physicochemical properties and most likely different functions.^29^ In recent studies, the occurrence of ether-linked phospholipids in farm animal tissues has been described; however, the physiological effects on the muscle of pigs fed with different PUFA-based diets were not investigated.^13,15^ Generally, plasmalogens are doubted to function as endogenous antioxidants in tissues.^29^ We assume that ether-linked phospholipids in the microalgae group may have been consumed to maintain antioxidant status/membrane homeostasis in the muscle. However, the complex interaction of lipid synthesis pathways and nutritional intervention is interconnected by lipid signaling events and crosstalk with other lipid classes.^29^ We likely detect effects of ether lipids signaling and PUFA-containing PLs that are known precursors of signaling molecular species like lipid mediators.^29^ This should be considered in further dietary lipid studies on pigs to cover such secondary up- or downregulation of other lipids occurring because of the primary effect of the diet.^15,30^ Based on the current knowledge, plasmalogens should play an important role in the regulation of membrane homeostasis, in particular membrane trafficking.^30^

Microalgae-supplemented diet led to increased concentrations of LPC 20:5, LPI 20:5, and LPI 22:6 in pig muscle and reduced concentrations of *n*-6 PUFA-containing lysophospholipid species (LPC 22:4, LPI 22:4). The overall low concentration level of these lysolipids points rather to changed lipid signaling than structural changes. Lysolipids are products of phospholipase A2 (PLA2), and for instance, LPC 22:6 shows anti-inflammatory properties compared to other *n*-6 PUFA-substituted LPCs.^31−33^ It is also noteworthy that despite the modifications in the acyl groups of individual species of LPC, LPE, and LPI in response to the microalgae diet, there was no significant difference in the sum concentrations of these PL classes between both diets groups (Table 1). This observation suggests that the functional properties of individual species of LPE, LPC, and LPI are potentially modulated by the incorporation of long-chain *n*-3 PUFAs, however without affecting sum PL class concentrations in pig muscle.

The DHA/EPA incorporation in membrane lipid species in pig muscles should originate almost completely from microalgae supplementation, although pigs are able to synthesize these long-chain *n*-3 PUFA de novo. Our recent results of fatty acid analysis of pig muscle tissues confirmed the high accumulation rate of DHA/EPA showing significantly higher total DHA and EPA concentrations in microalgae group, for DHA (97.4 mg/100g muscle) compared to the control group (14.7 mg DHA/100g muscle).^8^ However, it is not possible to infer the origin of DHA and EPA from microalgae supplementation or de novo synthesized using the available data. A number of pig diet intervention studies revealed that feeding of vegetable-based PUFA-rich supplements—lipids high in 18:3*n*-3 and 18:2*n*-6 (linseed or rapeseed, cakes/oils/meals)—did not result in enhanced DHA/EPA accumulation in muscle tissues.^34,35^ One reason seems to be that *n*-3 PUFA supplementation of pig diet inhibits the expression of transcription factors and genes encoding lipogenic enzymes (SREBP-1, ELOVL5, FADS1, and FADS2) and caused the endogenous de novo synthesis of DHA comprising lipids to remain unchanged.^36^ In particular, dietary *n*-3 PUFAs have been shown to inhibit the transcription of lipogenic genes by suppressing SREBP-1c gene expression in porcine muscle and adipose tissue.^25,26^ Based on current knowledge, it appears that *n*-3/*n*-6 PUFAs act as modulators for nuclear receptors and consequently suppress de novo fatty acid synthesis, so lipogenesis and *n*-3 PUFAs appear to be more effective than *n*-6 PUFAs.^27^ A recent pig dietary intervention study applying supplements of 5–10% insect meal in the diet indicated decreased incorporation of de novo synthesized DHA into hepatic phospholipid species using a lipidomic approach; however, no specific investigation into the skeletal muscle tissues was performed.^13^ For the endogenous synthesis of DHA, two pathways are assumed, one is the elongation of 22:5*n*-3 (DPA) to 24:5*n*-3 subsequently desaturated into 24:6*n*-3 (Δ6 desaturase) and backconverted into 22:6*n*-3 (DHA) via peroxisomal β-oxidation.^37^ The second potential pathway can be the direct desaturation of 22:5*n*-3 into 22:6*n*-3 (Δ4 desaturase).^38^ Thus, further detailed lipidomic studies combined with transcriptomic and proteomic studies on pigs fed with different diets are necessary to investigate the putative pathways of DHA incorporation into skeletal muscle tissues.

In conclusion, lipidomic analysis can improve our understanding of lipid metabolism and its influence on skeletal muscle physiology. Further investigation of dietary *n*-3 PUFA incorporation into storage and membrane lipids is required to gain insight into functional and physiological consequences of microalgae supplementation and other *n*-3 PUFA natural sources.

## Supporting Information Available

(XLSX)
Details on the internal standard mixture used in lipidomics analysis (S1), concentration of all lipid species in muscles of both diet groups considered in the statistical analysis (S2), profiles of phosphatidylethanolamine—plasmalogens (mol percentage) (S3), and exemplary MS spectra of TAGs of muscle tissue lipid extracts in the positive ion mode (S4) (PDF)
