# Supplementary material for: Are n-3 PUFAs from Microalgae Incorporated into Membrane and Storage Lipids in Pig Muscle Tissues?—A Lipidomic Approach
Source: ACS Omega. 2022 Jul 8;7(28):24785–94. doi: 10.1021/acsomega.2c02476 (PMC9301695; doi:10.1021/acsomega.2c02476)
Supplement: Supplementary file 2 — ao2c02476_si_002.pdf [file ao2c02476_si_002.pdf]

Are n-3 PUFA from microalgae incorporated into membrane- and storage lipids in pig muscle tissues? – A lipidomic approach

Dirk Dannenberger[1*,a], Anja Eggert[2,a], Claudia Kalbe[1], Anna Woitalla[3] and Dominik Schwudke [3,4,5]

[1]Research Institute for Farm Animal Biology, Institute of Muscle Biology and Growth, Lipid Metabolism and muscular Adaptation workgroup, 18196 Dummerstorf, Germany, dannenberger@fbn-dummerstorf.de, kalbe@fbn-dummerstorf.de

[2]Research Institute for Farm Animal Biology, Institute of Genetics and Biometry, 18196 Dummerstorf, Germany, eggert@fbn-dummerstorf.de

[3]Research Center Borstel – Leibniz Lung Center, Division of Bioanalytical Chemistry, 23845 Borstel, Germany, dschwudke@fz-borstel.de, awoitalla@fz-borstel.de

[4]German Center for Lung Research (DZL), Airway Research Center North (ARCN), Research Center Borstel - Leibniz Lung Center, 23845 Borstel, Germany, dschwudke@fz-borstel.de

[5]German Center for Infection Research, Thematic Translational Unit Tuberculosis, Research Center Borstel- Leibniz Lung Center, 23845 Borstel, Germany, dschwudke@fz-borstel.de

The supporting Information includes the file (Supplemented figure 1.pptx)

S4 Exemplary MS spectra of muscle tissue lipid extracts in the positive ion mode
S5 Exemplary MS^2 spectra of TAG 52:2 present in muscle tissue lipid extracts
S6 Exemplary MS^2 spectra of TAG 56:6 present in muscle tissue lipid extracts
S7 Exemplary MS^2 spectra of TAG 54:6 present in muscle tissue lipid extracts

Supplement Figure 1

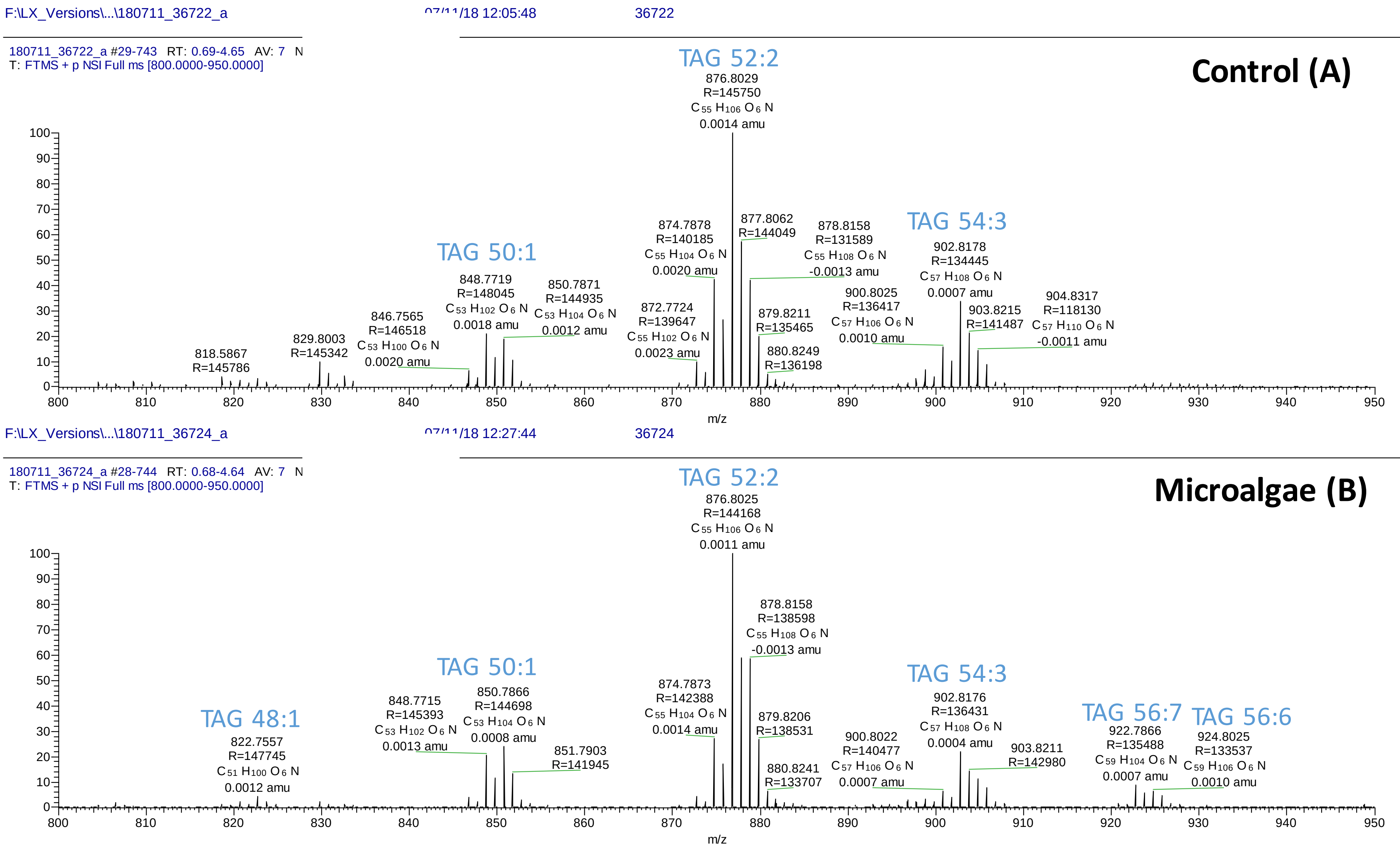

S4 Exemplary MS spectra of muscle tissue lipid extracts in the positive ion mode. TAGs are ionized as $[M+NH_4]^+$ adduct ions.
The *m/z* region 800-950 is shown for A) control diet B) diet supplemented with microalgae.
Main TAG species are assigned including *m/z*, resolution (R), chemical sum composition and mass difference.

Supplement Figure 2

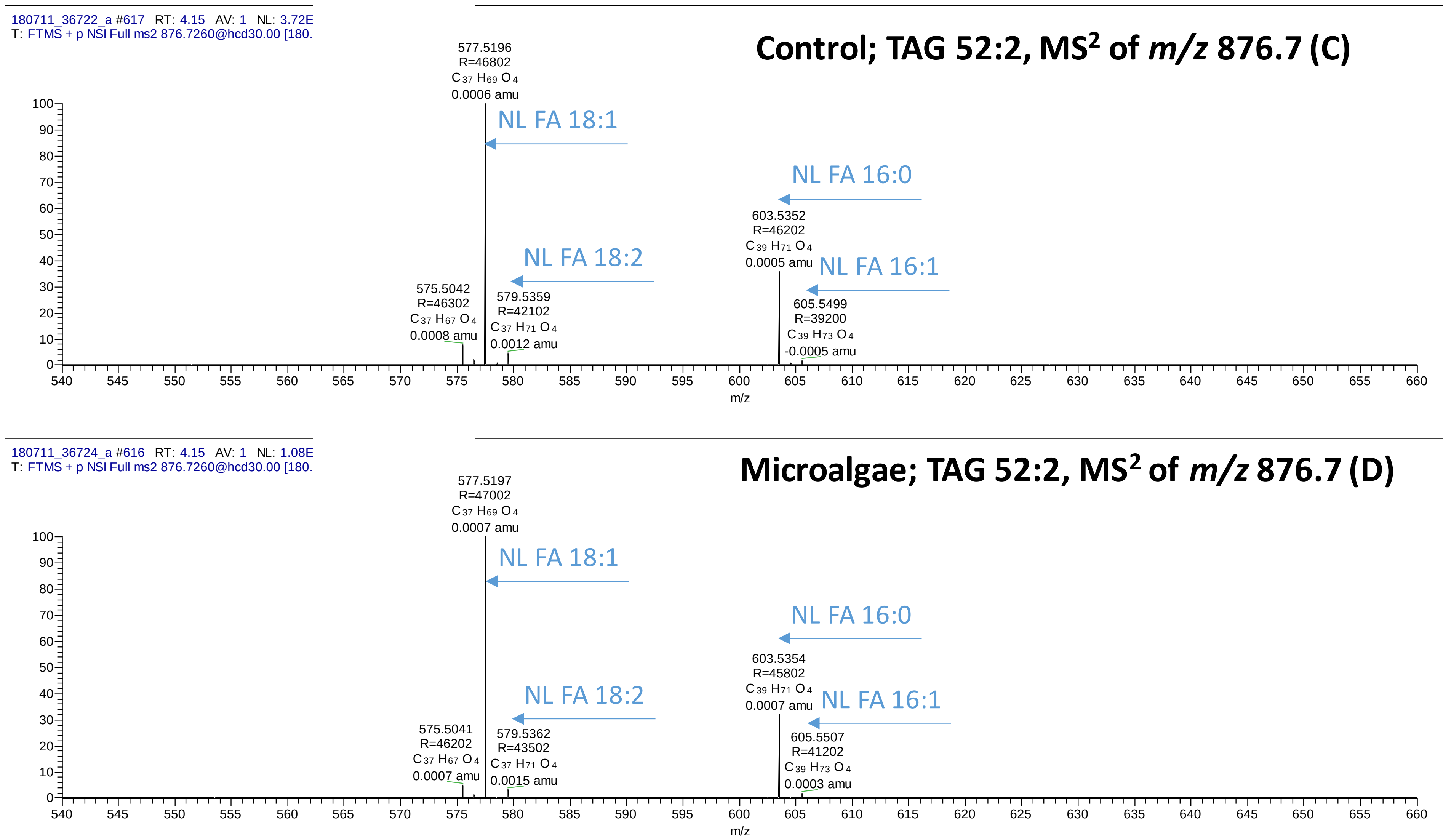

S5 Exemplary MS^2 spectra of TAG 52:2 present in muscle tissue lipid extracts C) control diet D) diet supplemented with microalgae. Major peaks are assigned according to earlier described fragmentation of $[TAG+NH_4]^+$ adduct ions that undergo a neutral loss (NL) of the fatty acid and ammonia [35]. The spectral region (*m/z* 540-660) is shown for better overview on the resulting $[DAG\text{-}OH]^+$ fragment ions.

Supplement Figure 3

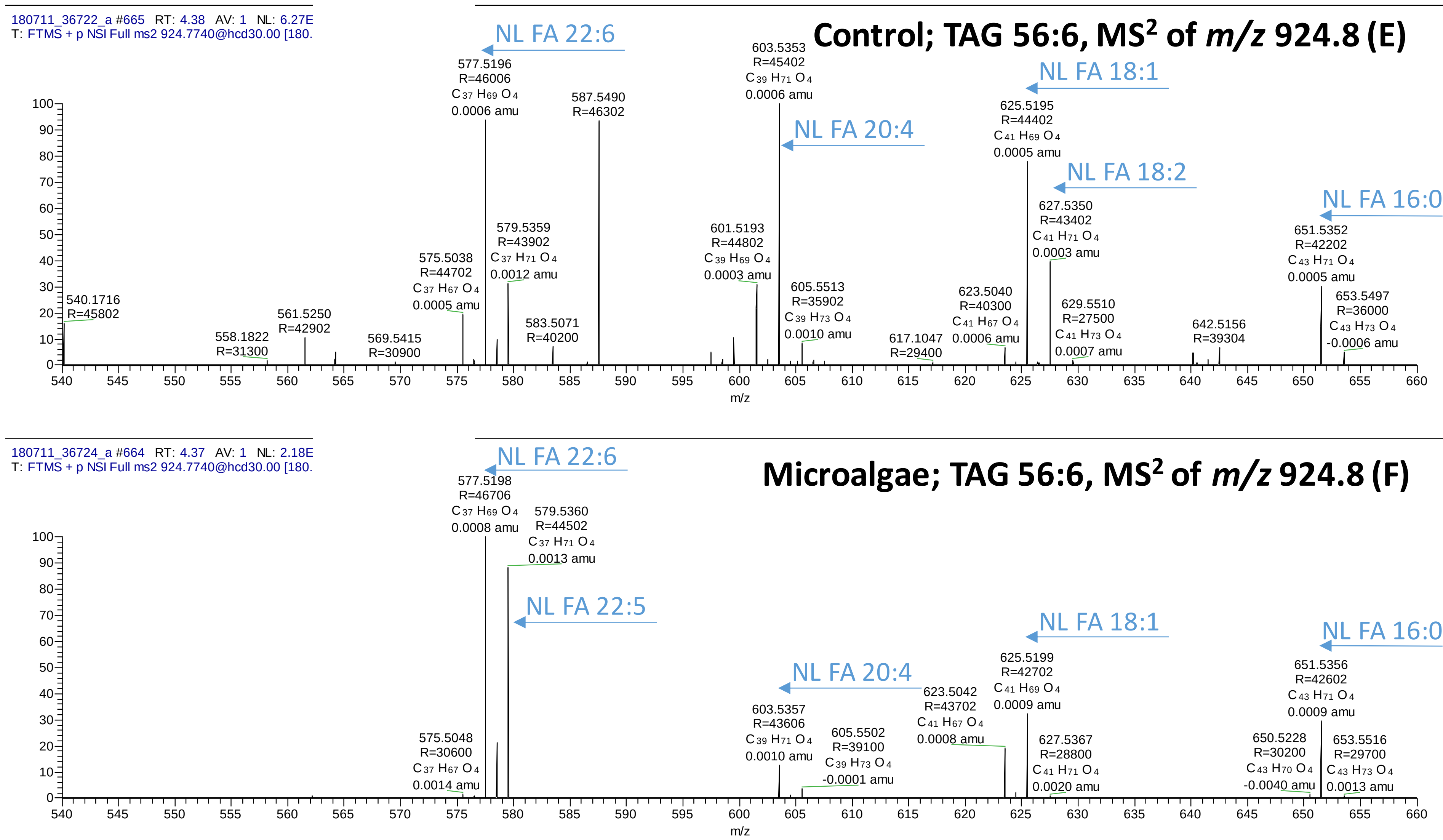

S6 Exemplary MS^2 spectra of TAG 56:6 present in muscle tissue lipid extracts E) control diet F) diet supplemented with microalgae. Major peaks are assigned according to earlier described fragmentation of $[TAG+NH_4]^+$ adduct ions that undergo a neutral loss (NL) of the fatty acid and Ammonia [35]. The spectral region (*m/z* 540-660) is shown for better overview on the resulting $[DAG\text{-}OH]^+$ fragment ions.

Supplement Figure 4

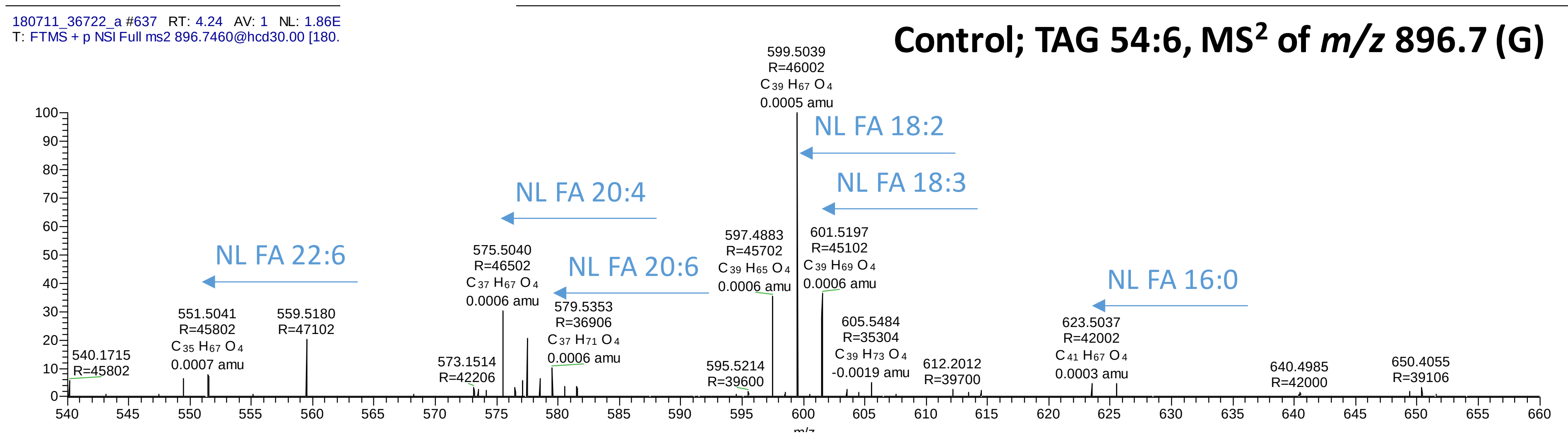

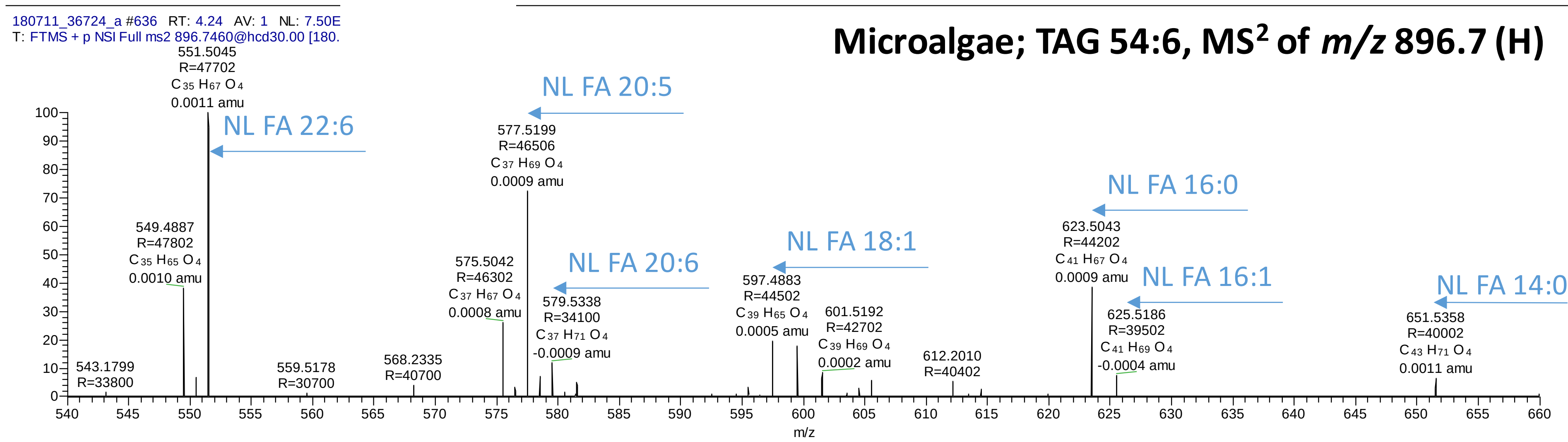

S7 Exemplary MS² spectra of TAG 54:6 present in muscle tissue lipid extracts G) control diet H) diet supplemented with microalgae. Major peaks are assigned according to earlier described fragmentation of $[TAG+NH_4]^+$ adduct ions that undergo a neutral loss (NL) of the fatty acid and ammonia [35] The spectral region (*m/z* 540-660) is shown for better overview on the resulting $[DAG\text{-}OH]^+$ fragment ions.
